# Circulating CD4+, CD8+, and double-negative T cells in ischemic stroke and stroke-associated infection: a prospective case-control study

**DOI:** 10.3389/fncel.2025.1547905

**Published:** 2025-04-24

**Authors:** Magdalena Telec, Magdalena Frydrychowicz, Radosław Kazmierski, Izabela Wojtasz, Grzegorz Dworacki, Wojciech Kozubski, Maria Łukasik

**Affiliations:** ^1^Department of Neurology, Poznan University of Medical Sciences, Poznan, Poland; ^2^Department of Immunology, Poznan University of Medical Sciences, Poznan, Poland; ^3^Department of Neurology, Collegium Medicum, University of Zielona Gora, Zielona Gora, Poland

**Keywords:** ischemic stroke, stroke associated infection, CD4+ T cells, CD8+ T cells, CD4+/CD8+ratio, double negative T cells

## Abstract

**Introduction:**

Adaptive immunity after a stroke results in a shift of T cells between compartments, leading to peripheral lymphopenia and an increased number of T cells within the brain lesion. Stroke-associated infection (SAI) presents a clinically significant challenge in stroke units. The role of T-cell subsets in the post-stroke immune response and in SAI remains unclear. Thus, we aimed to observe the quantitative changes of circulating CD4+, CD8+, double-negative T cells, and the CD4+/CD8+ ratio in stroke and SAI.

**Methods:**

We prospectively assessed circulating CD4+, CD8+, and double-negative T cells using flow cytometry in 52 patients on days 1, 3, 10, and 90 after ischemic stroke. We compared the results to those obtained from age-, sex-, and vascular risk factor-matched controls. We analyzed lymphocyte parameters in relation to clinical outcome, SAI, infarct lesion volume, and risk factor burden.

**Results:**

There were no differences in the studied parameters between stroke patients and controls, as well as between subjects with and without SAI. A higher percentage of CD4+ T cells and a higher CD4+/CD8+ ratio correlated with better clinical status in the acute and subacute phases, while CD8+ T cells showed the opposite correlation. The percentage of CD8+ T cells positively correlated with CRP levels during the acute and subacute phases of stroke, as well as in the control group. A negative correlation was noted between the percentage of CD4+ T cells on D1 and the serum CRP level on D10 after stroke. Similarly, the CD4+/CD8+ ratio on D1 negatively correlated with CRP on D1, D3, and D10. In patients with a history of hypertension (HT), there was a higher percentage of CD8+ T cells and a lower percentage of CD4+ T cells in the acute phase of stroke than those without HT.

## 1 Introduction

Stroke-induced immunodepression syndrome, characterized by lymphopenia, contributes to the increased risk of infection in patients hospitalized due to ischemic stroke. The likelihood of pneumonia or urinary tract infection in these patients ranges from 21 to 65% (Chamorro et al., 2007). Thirty percent of patients with stroke-associated infections (SAI) will die, highlighting the clinical significance of SAI. The immunological reaction to stroke is mostly concerned with the adaptive response (Meisel et al., 2005).

T-lymphocytes are the main representatives of adaptive response cells in the central nervous system (Giunti et al., 2003). T cells circulate in the cerebrospinal fluid of healthy individuals, constantly invigilating the central nervous system (De Graaf et al., 2010; Ransohoff et al., 2003). CD4+ and CD8+ T cells make up the majority of T-lymphocytes. The proportion of CD4+ and CD8+ T cells in cerebrospinal fluid is 3.5:1, and some are memory cells (Giunti et al., 2003; Stuve et al., 2006). CD4+ T cells mediate immune response by secreting specific cytokines and activating B cells, cytotoxic T cells, and non-immune cells, and also play a critical role in the suppression of immune reaction. The subsets of CD4+ T cells include classical T-helper 1 (Th1) and T-helper 2 (Th2) cells, T-helper 17 (Th17), follicular helper T cells (Tfh), induced T-regulatory cells (iTreg), and regulatory type 1 cells (Tr1) and possibly distinct T-helper 9 (Th9) (Luckheeram et al., 2012). Th1 cells are involved in the elimination of intracellular pathogens and are associated with organ-specific autoimmunity. In contrast, Th2 cells are involved in immune responses against extracellular parasites and play a major role in asthma and other allergic diseases (Del Prete, 1992). CD8+ T cells are crucial for immune defense against intracellular pathogens, including viruses and bacteria, as well as for tumor surveillance. CD8+ T cells activated by antigens from infected or malignant cells present a cytotoxic ability, releasing perforin and granzymes or eliminating target cells through Fas/FasL interactions (Catalfamo and Reali, 2020).

CD4−CD8− T cells, called double-negative T cells (DNTs), constitute a small subpopulation of peripheral T cells (Brandt and Hedrich, 2018). The origin and function of DNT cells are still unclear. In healthy individuals, they form a rare and heterogeneous T-cell subset (1–5% of the total T-cell population). However, the number of DNT cells increases in various inflammatory conditions, where they exhibit different effector phenotypes and infiltrate inflamed tissues (Brandt and Hedrich, 2018; Doyle et al., 2015).

Since the brain is no longer considered a site of immune privilege, despite the presence of the blood–brain barrier and lack of conventional lymphatic vessels, many immune cells can infiltrate the central nervous system and may contribute to immune surveillance in the brain. After an ischemic stroke, T and B cells, together with macrophages and neutrophils, infiltrate the brain and interact with the glial cells (Hersh and Yang, 2018). While the number of T cells decreases peripherally, at the same time it increases in the ischemic brain lesion. Intriguingly, it happens within the first 24 h and can persist for a longer period (Gelderblom et al., 2009). The extent of the infiltration of T cells varies among stroke models (Gill and Veltkamp, 2016). A significant increase in CD4+ and CD8+ T cells was seen in human ischemic infarct lesions, with the dominant population of CD8+ T-cells (Zrzavy et al., 2018). The experimental stroke model showed infiltration of CD8+ T cells in an ischemic area within 3 h since vessel occlusion and CD4+ T cells during the first 24 h (Grønberg et al., 2013). Other research revealed infiltration of CD4+ and CD8+ T cells on day 3 (Stevens et al., 2002), day 5 (Liesz et al., 2011), or even 7 weeks after stroke onset (Doyle et al., 2015). In mice with stroke, CD4+ T cell accumulation was noticed for a 30-day period, with a peak on the 14th day of stroke (Gill and Veltkamp, 2016). It shows that antigen-specific reactions to brain ischemia can take place in acute, subacute, and chronic phases. Furthermore, the accumulation of DNT was observed in the area of the ischemic hemisphere in 1–3 days after reperfusion, which suggested an important role of these cells in inflammation (Gelderblom et al., 2009; Meng et al., 2019). Despite these findings, it is still unclear whether the T cells that infiltrated the brain during acute post-stroke neuroinflammation support or hinder functional recovery (Selvaraj and Stowe, 2017). It has been reported that the adaptive transfer of lymphocytes against myelin antigen exacerbates stroke lesions and that proinflammatory lymphocytes are detrimental during an early stage of ischemic brain injury (Jin et al., 2018). Transgenic animals deficient in CD4+ or CD8+ T cells consistently have smaller infarcts in different stroke models (Liesz et al., 2009a; Liesz et al., 2011; Kleinschnitz et al., 2010; Yilmaz et al., 2006). Moreover, antibody-mediated depletion of CD4+ and CD8+ T cells reduced infarct volume and improved functional outcome, respectively (Mracsko et al., 2014; Gelderblom et al., 2012; Shichita et al., 2009). Antigen-specific activation of cytotoxic CD8+ T cells leads to targeted neuronal cell death by close intercellular interaction and via the perforin–granzyme pathway (Mracsko et al., 2014).

Although ischemic stroke reduces the numbers of lymphocytes in the circulation and lymphoid organs (Prass et al., 2003), subjects with acute ischemic stroke had a significantly higher number of circulating CD4+ T cells in peripheral blood compared to control subjects without acute ischemic lesions (Tuttolomondo et al., 2015). There are also qualitative shifts, and among dominating CD4+ T cells, type Th2 prevails more than Th1. The goal of the response with the domination of Th2 type, a source of anti-inflammatory cytokines, is probably neuroprotection and minimization of stroke lesions (Zhang et al., 2018). One of the SAI risk factors, next to older age, severe clinical status on admission, and large size of infarct lesions, is a decreased number of CD4+ T cells in the initial phase of stroke (Jiang et al., 2017).

Considering the biological role of peripheral immunosuppression and questioning whether it diminishes the deleterious effect of post-stroke systemic inflammation, we aimed to observe the quantitative changes in CD4+ T cells, CD8+ T cells, and DNT, as well as the CD4+/CD8+ ratio, in stroke and SAI. We also considered factors such as stroke lesion volume, neurological status, and comorbidities, particularly those related to vascular disease risk factors.

## 2 Materials and methods

### 2.1 Subjects

We studied 52 patients with acute ischemic stroke and 34 control subjects. Patients were recruited from the Stroke Unit at the Department of Neurology and Cerebrovascular Disorders of Poznan University of Medical Sciences, Poznan, Poland, between November 2018 and May 2019. A total of 161 consecutive patients admitted with suspected stroke were prospectively screened. The inclusion criteria were: ischemic stroke, symptom onset within 24 h before clinical evaluation and blood sampling, and age over 40. The clinical diagnosis of ischemic stroke was confirmed based on radiological evidence in cranial computed tomography (CT) scans and/or cranial MRI at admission. Patients with TIA, intracerebral hemorrhage, malignancies, autoimmune diseases, past/acute coronary syndrome, and/or peripheral artery disease within 12 months preceding entry into the study, hematological disorders, liver and renal failure, alcohol, and/or drug abuse, and a history of infection and/or the use of antibiotics and/or immunosuppressants and/or steroids within the preceding 3 months were excluded (Supplementary material 1). Physical and neurological examinations were performed in the acute phase of the stroke on days 1 (D1) and 3 (D3), in the subacute phase on day 10 (D10), and in the convalescent phase, 90 ± 3 days (D90) after stroke. To measure stroke severity, the Scandinavian Stroke Scale (SSS) was used (on the SSS the higher the score, the lesser the neurological deficit) (Table 1). The etiology of the stroke was classified according to the TOAST classification. The diagnostic measures included blood pressure and BMI (individual’s weight in kilograms divided by the square of height in meters). The laboratory investigations include blood counts with automatic smear tests, biochemical, coagulation, and urine tests. Clinical and laboratory assessments were supplemented by chest radiographs, ECG, color Doppler duplex ultrasonography of cervical and vertebral arteries, transcranial Doppler ultrasonography (TCCD), and transthoracic echocardiography. Imaging of the brain with MRI was performed within 24 h from stroke onset and 90 ± 3 days after the stroke (Table 2). We also documented medical history details, including the presence of hypertension, diabetes mellitus, hypercholesterolemia, atrial fibrillation, heart ischemic disease, obesity, smoking, previous strokes, and use of acetylsalicylic acid, anticoagulants, statins, hypotensive drugs, oral diabetics, and non-steroid anti-inflammation drugs (Table 3). All stroke patients were assessed for SAI on day 10 after the stroke. SAI was diagnosed when the following criteria were met: (1) body temperature > 37.8°C in a patient with symptoms suggestive of infection; (2) white blood cell (WBC) count >11,000/mL or <4,000/mL; (3) inflammatory lesions in the chest X-ray; (4) blood or urine culture positive for pathogen; and/or (5) antibiotic or chemotherapeutic therapy, all within 7 days of the onset of stroke symptoms.

**Table 1:** Scandinavian stroke scale.

Scandinavian stroke scale
Prognostic score (pSSS)
1. Consciousness fully conscious somnolent, can be awakened to full consciousness reacts to verbal commands, but is not fully conscious
2. Eye movement no gaze palsy gaze palsy present conjugate eye deviation
3. Arm, motor power raises arm with normal strength raises arm with reduced strength raises arm with flexion in the elbow can move but not against gravity paralysis
4. Leg, motor power raises straight leg with normal strength raises straight leg with reduced strength raises leg with flexion of the knee can move, but not against gravity paralysis
Long term score (dSSS)
1. Arm, motor power—like in the prognostic score
2. Hand, motor power normal strength reduced strength in full range some movement, fingertips do not reach the palm paralysis
3. Leg motor power—like in the prognostic score
4. Orientation correct for time, place, and person two of these one of these completely disoriented
5. Speech no aphasia limited vocabulary or incoherent speech more than yes/no but no longer sentences only yes/no or less
6. Facial palsy none/dubious present
7. Gait walks 5 m without aids walks with aids walks with the help of another person sits without support bedridden/wheelchair

**Table 2:** List of research procedures at specific time points.

	Stroke D1	Stroke D3	Stroke D10	Stroke D90
Medical interview	x			
Treatment	x	x	x	x
Neurological examination	x	x	x	x
Stroke classification (TOAST, OCSP)	x			
SSS	x	x	x	x
Body weight, height	x			x
Vital signs	x	x	x	x
ECG (12 lead)	x	x		
Morphology with smear	x	x	x	x
Biochemistry	x			x
CRP	x	x	x	x
urinalysis	x	x	x	
SAI assessment	x	x	x	
Flow cytometry	x	x	x	x

**Table 3:** Baseline characteristics of the studied groups.

	Stroke patients, D1 (*n* = 52)	Disease controls (*n* = 34)	*p*_W1 vs. DC_
Age, years	69 (±12.1)	68 (±12.7)	*p* = 0.658
BMI (kg/m^2^)	25.4 (23.6–28.4)	27.0 (24.9–30.6)	*p* = 0.368
Female subjects, *n* (%)	25 (48.1%)	13 (38.2%)	*p* = 0.386
Hypertension, *n* (%)	42 (80.8%)	30 (88.2%)	*p* = 0.551
Diabetes, *n* (%)	10 (19.2%)	6 (17.7%)	*p* > 0.99
Ischemic heart disease, *n* (%)	19 (36.5%)	14 (41.2%)	*p* = 0.821
Atrial fibrillation, *n* (%)	20 (38.5%)	3 (8.8%)	*p* = 0.003
Smoking, *n* (%)	18 (34.6%)	7 (20.6%)	*p* = 0.225
Hyperlipidemia, *n* (%)	16 (30.8%)	12 (35.3%)	*p* = 0.814

The disease control (DC) group consisted of 34 subjects matched for age, sex, and vascular disease risk factors. DC subjects had at least two vascular disease risk factors but had never experienced clinical symptoms of acute cerebral, coronary, or peripheral ischemia. Disease controls underwent the same laboratory tests and procedures as stroke patients except for chest radiograph, ECG, color Doppler duplex ultrasonography of cervical and vertebral arteries, transcranial Doppler ultrasonography (TCCD), transthoracic echocardiography, and blood sampling, which was performed only once.

The study protocol was approved by the bioethics committee of the Poznan University of Medical Sciences (no. 139/2018). Informed consent was obtained from all study subjects.

### 2.2 Flow cytometry

To detect the absolute number and percentage of CD4+ and CD8+ T cells and CD4−CD8− T cells (DNT), flow cytometry combined with counting was used. In the stroke group, blood samples were collected within 24 h of symptoms onset (D1—the first wave of immune activation), as well as on days 3 (D3—early inflammatory window), 10 (D10—peak in adaptive immune response), and 90 ± 3 (D90—long-term immune changes) after the stroke. In the DC group, blood samples were taken once. Cell immunophenotyping was performed using flow cytometry with the direct fluorescence method. The cells were stained with combinations of the following antibodies: anti-CD3 APC-Cy7, anti-CD4 PE-Cy7, and CD8 PerCp. Test tubes with 15 μL of anti-CD3/CD4/CD8 cocktail combined with 100 μL of peripheral blood, previously sampled with K2EDTA (ethylenediaminetetraacetic acid dipotassium salt) in vacutainer tubes, were gently mixed by a vortex and incubated at room temperature in the dark for 20 min. Then, 500 μL lysis buffer (FASC lysing solution, Becton Dickinson, USA) was added to each test tube, and the tubes were incubated for 10 min. Lysis was stopped when the phosphate-buffered saline solution (PBS, Roche Diagnostic, Germany) was added. The samples were washed by spinning at 250 × *g* for 4 min to separate residually lysed erythrocytes, plasma proteins, and other blood elements from leukocytes. This step was performed twice. The cell pellets were resuspended in 200 μL of PBS. Data acquisition and analysis were performed using the FACSCantoTM II flow cytometry system and FACS DivaTM software (BD Biosciences) with a standard 6-color filter configuration. Lymphocytes were identified based on cell characteristic properties in the forward (FSC) and side (SSC) scatter. For additional analyses, gates were restricted to CD3+, CD3+CD4+CD8+, and CD3+CD4−CD8−. For each examined antibody, the percentage of positive cells and mean fluorescence intensity (MFI) were determined.

### 2.3 Infarct volume measurement

To assess infarct lesion volume, MRI sequences on a Siemens, AVANTO 1.5 T, Erlangen, Germany clinical scanner were performed. The standard imaging protocol included: FLAIR, T2-weighted images, diffusion-weighted image (DWI) sequencing with an increasing b-factor, ADC map, and T1-weighted images. Recent hyperintense ischemic lesions were traced according to the segmentation method from DWI with *b* = 1,000 s/mm^2^. The measured surface area was multiplied by the thickness of the layers within the visualized ischemic region. In the follow-up MRI examination, performed after 90 ± 3 days, the volume of the lesion was determined using the same method, but in FLAIR sequences. The lesion volume measurement was expressed in milliliters (mL). The typical imaging parameters were as follows: DWI: *b* = 0–500–1,000–2,000 s/mm^2^, TR/TE ≈ 4700/113 ms; FLAIR: ≈9,000/86 ms; TI ≈ 2,200 ms; FOV = 24 cm, matrix = 256 × 256; NEX = 1; and base resolution = 256, where: TR—repetition time, TE—echo time, TI—inversion time, FOV—field of view, NEX—number of acquisitions. The concordance of the intra-rater readings was 89.1% [*κ* = 0.76; 95% confidence interval (CI) 0.64–0.83 for DWI and FLAIR sequences].

### 2.4 Statistical analyses

Statistical analyses and database management were performed with the software system Statistica, version 13 (Statistica, 2017), and GraphPad Prism, version 8.0.1 (GraphPad Software, San Diego, CA, USA). The sample size was evaluated *a priori* with standard statistical criteria for the sample size estimation and statistical power. For the data with a normal distribution, the Shapiro–Wilk and Kolmogorov–Smirnov tests were used. The non-normal distribution data were analyzed using non-parametric methods and presented as median values and interquartile range values. Normal distribution data were presented as means ± SD. For comparisons of normally distributed data, the parametric Student’s t-test, ANOVA, and *post-hoc* Tukey’s test were used. Multiple comparisons within the stroke group were performed using the Wilcoxon signed-rank test with Bonferroni correction; differences were assumed to be significant at a *p*-value of <0.01. To compare data between patients and the control group, the Mann–Whitney U-test was used. Categorical data were compared using the chi-squared test or Fisher’s exact test, where appropriate. The Spearman rank correlation test was used to test for possible relationships between the studied parameters. A *p*-value of <0.05 was assumed to be significant, where not indicated otherwise.

## 3 Results

We analyzed T cells in the peripheral blood of 52 stroke patients (48% were female), with a mean age of 69 ± 12 years. Table 1 shows the demographic and clinical characteristics. On D3, 47 subjects were evaluated—5 patients refused to continue blood sampling; on D10, 45 subjects were evaluated—1 more patient declined to participate in the follow-up visit and 1 patient died; and on D90, 33 subjects were evaluated—11 patients refused to participate in the last follow-up visit due to disability and dependency or inpatient rehabilitation at that time and 1 patient died. The controls were matched with the stroke patient according to the vascular disease risk factors, but atrial fibrillation was more prevalent in the stroke group (42.2% vs. 0%, *p* < 0.0001) (Table 3). The median time from stroke onset to blood sampling on D1 was 17 h (12–22).

### 3.1 Kinetics of circulating lymphocytes

**Figure 1:**
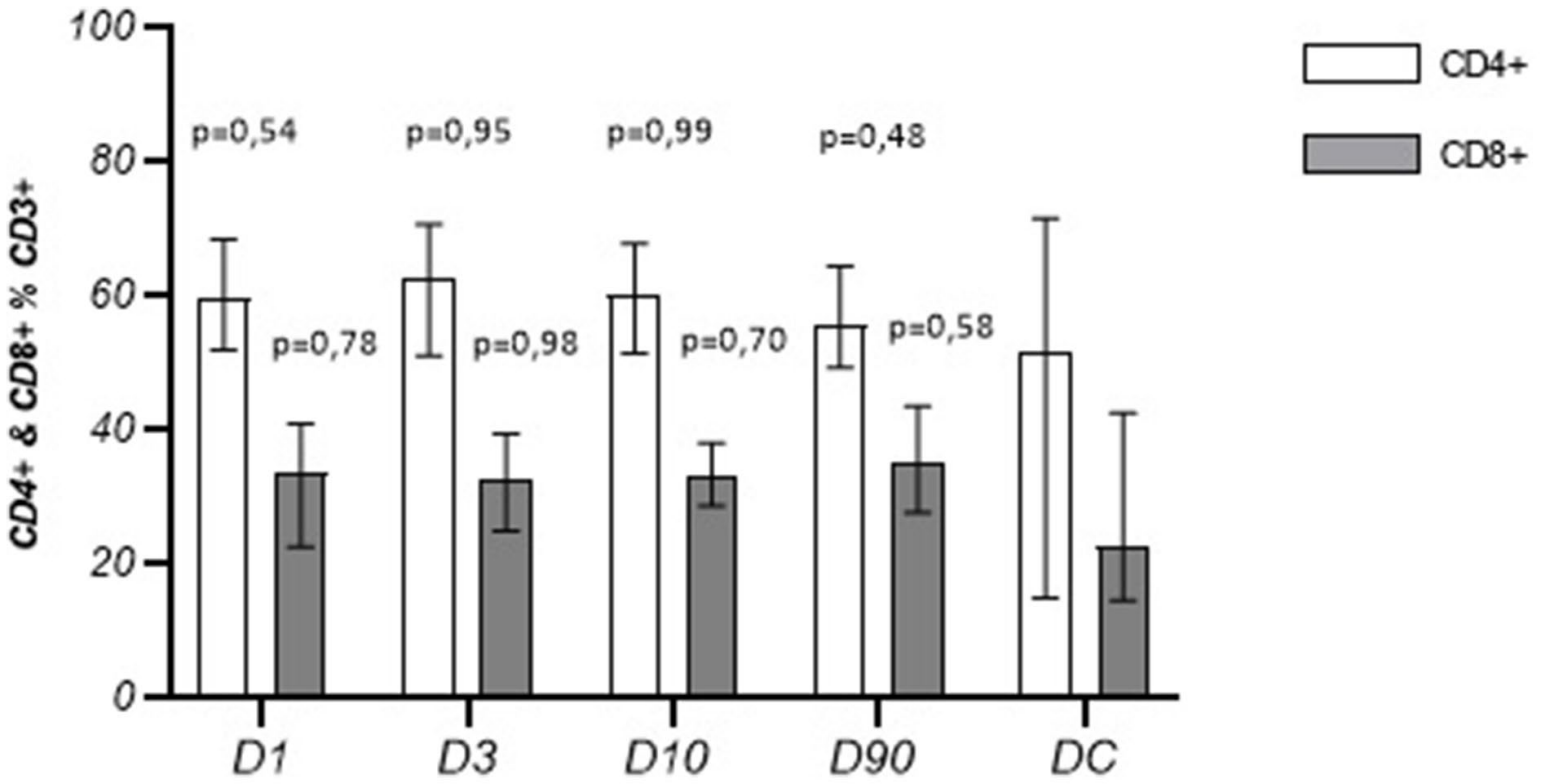
Percentage of CD4+ and CD8+ lymphocytes in the following stroke days in comparison to the disease control group (Me ± QR).

**Figure 2:**
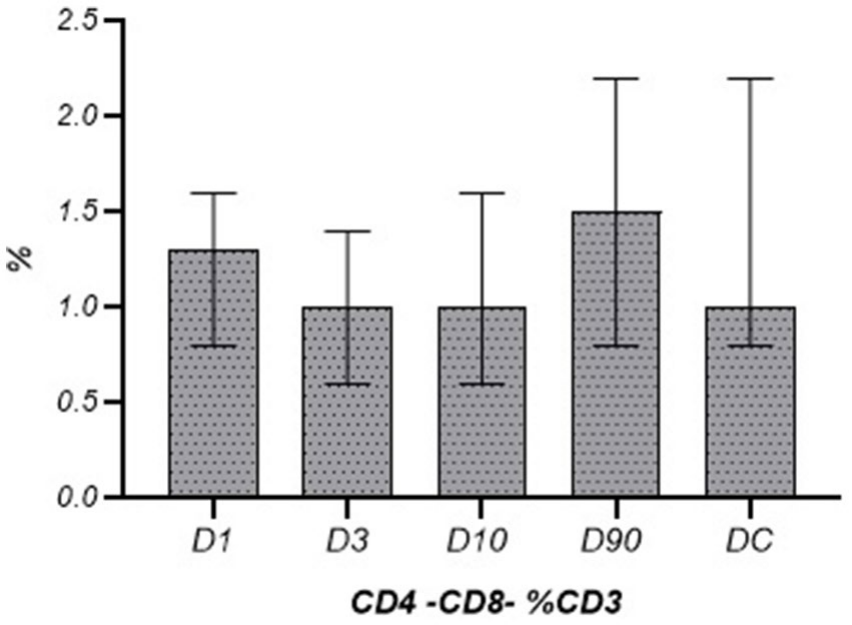
Percentage of CD4−CD8− lymphocytes in the following stroke days in comparison to the disease control group (Me ± QR).

**Figure 3:**
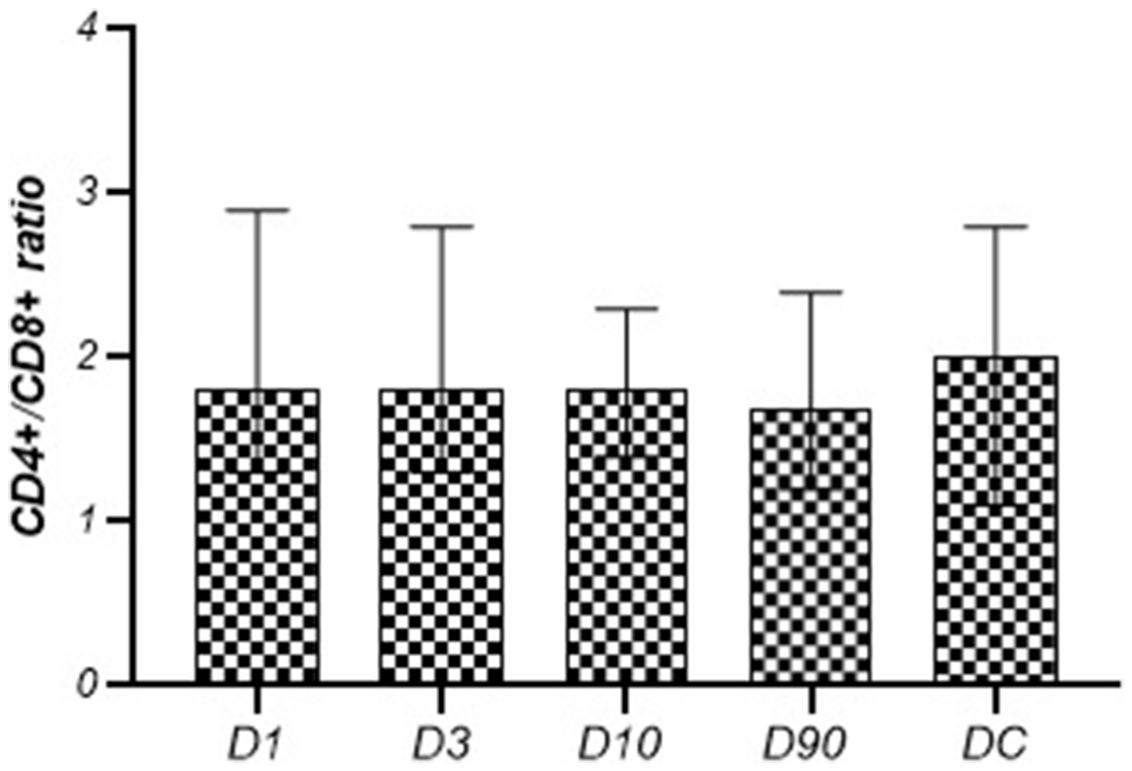
CD4+/CD8+ ratio in the following stroke days in comparison to the disease control group.

The absolute number and the percentage of T cells on post-stroke D1, D3, and D10 were lower than those observed in the controls, and on D1 the percentage of T cells was significantly lower than that measured on D90 (Table 4). The peripheral blood leukocyte count (WBC), the absolute number, and the percentage of CD4+ and CD8+ T cells in the population of CD3+ lymphocytes or CD4−CD8− T cells in the population of CD3+ lymphocytes did not change significantly during the time after the stroke. The values did not differ significantly from those observed in the control group either (Table 5; Figures 1, 2). The CD4+/CD8+ ratio did not change significantly at the time after stroke or when compared to the control group (Figure 3).

**Table 4:** Prospective quantitative analysis of white blood cell subpopulations in stroke patients.

	Stroke D1	Stroke D3	Stroke D10	Stroke D90	Disease controls	*p*_D1 vs. DC_	*p*_D3 vs. DC_	*p*_D10 vs. DC_	*p*_D90 vs. DC_
WBC ×10^3^/μL	7.4 (5.8–10.6)	7.6 (5.7–10.2)	7.2 (5.9–8.1)	6.8 (5.6–7.7)	6.8 (5.7–8.4)	*p* = 0.119	*p* = 0.281	*p* = 0.816	*p* = 0.448
Lymphocytes ×10^3^/μL	1.7 ± 0.9	2.0 ± 0.9	1.8 ± 0.7	1.9 ± 0.7	2.3 ± 1.2	***p* = 0.008**	*p* = 0.114	***p* = 0.024**	*p* = 0.075
Lymphocytes %	21.5 ± 11.6	25.3 ± 10.9	25.8 ± 10.2	28 ± 7.5	31.3 ± 10	***p* = 0.000**	***p* = 0.018**	***p* = 0.029**	*p* = 0.155
Monocytes ×10^3^/μL	0.6 (0.5–0.8)	0.7 (0.5–1.0)	0.6 (0.5–0.8)	0.6 (0.5–0.8)	0.7 (0.6–0.8)	*p* = 0.255	*p* = 0.538	*p* = 0.652	*p* = 0.348
Monocytes %	8 (6.5–10)	10 (8–11)	9 (8–11)	9 (8–11)	9.5 (8–11.5)	***p* = 0.016**	*p* = 0.693	*p* = 0.344	p = 0.658
Neutrophiles ×10^3^/μL	4.7 (3.3–7.4)	4.0 (3.3–7.0)	4.2 (2.9–4.8)	4.1 (3.0–4.9)	4.1 (2.8–5.1)	*p* = 0.056	*p* = 0.127	*p* = 0.974	*p* = 0.875
Neutrophiles%	65 (55–75)	62 (53–72)	59.5 (50–65)	60 (54–63)	56 (51–65)	***p* = 0.009**	***p* = 0.047**	*p* = 0.610	*p* = 0.340
Eozynophiles ×10^3^/μL	0.1 (0.0–0.2)	0.2 (0.1–0.3)	0.2 (0.1–0.3)	0.2 (0.1–0.3)	0.2 (0.1–0.3)	***p* = 0.017**	*p* = 0.553	*p* = 0.484	*p* = 0.918
Eozynophiles %	1 (0–3)	3 (1–5)	3 (2–5)	3 (2–5)	3 (2–5)	**p = 0.003**	*p* = 0.784	*p* = 0.508	*p* = 0.747
RBC ×10^6^/μL	4.5 ± 0.5	4.5 ± 0.6	4.4 ± 0.6	4.4 ± 0.4	4.4 ± 0.6	p = 0.658	*p* = 0.684	*p* = 0.781	*p* = 0.731
PLT ×10^3^/μ×	231 ± 65.9	235 ± 79.4	254 ± 97	239 ± 59	245 ± 69	*p* = 0.379	*p* = 0.584	*p* = 0.679	*p* = 0.720

*p-values in bold are statistically significant.*

**Table 5:** Prospective quantitative analysis of white blood cell subpopulations in stroke patients.

	Stroke D1	Stroke D3	Stroke D10	Stroke D90	Disease control	*p*
CD4^+^ (cells/μL)	525 ± 240	611 ± 302	534 ± 241	523 ± 237	625 ± 364	*p*_D1 vs. DC_ = 0.147 *p*_D3 vs. DC_ = 0.863 *p*_D10 vs. DC_ = 0.212 *p*_D90 vs. DC_ = 0.204
CD8^+^ (cells/μL)	263 (158–343)	295 (198–447)	280 (192–366)	321 (175–425)	290 (158–547)	*p*_D1 vs. DC_ = 0.330 *p*_D3 vs. DC_ = 0.874 *p*_D10 vs. DC_ = 0.667 *p*_D90 vs. DC_ = 0.959

### 3.2 Lymphocyte subsets and clinical parameters

#### 3.2.1 Neurological status

To determine neurological deficits, patients were assessed on SSS. Significant differences were found between the clinical status of patients on D1 and at other studied time points. The lowest scores in SSS were on D1, while on D3 (*p* < 0.0001), D10 (*p* < 0.0001), and D90 (*p* < 0.0001)—the SSS score increased (*p* = 0.001). Moreover, a higher score in SSS was revealed on D90 compared to D10 (*p* = 0.003) and D3 (*p* = 0.026) (Table 6).

**Table 6:** Assessment of neurological deficit and degree of disability at designated time points.

	Stroke D1	Stroke D3	Stroke D10	Stroke D90
pSSS	20 (14–22) *p*_D1 vs. D3_ = 0.417 ***p***_**D1 vs. D10**_ **= 0.002**	21 (18–22) *p*_D3 vs. D10_ = 0.167 *p*_D3 vs. D90_ = −	22 (19–22) *p*_D10 vs. D90_ = −	–
dSSS	32 (18–41) ***p***_**D1 vs. D3**_ **< 0.0001** ***p***_**D1 vs. D10**_ **< 0.0001** ***p***_**D1 vs. D90**_ **< 0.0001**	42 (25–46) *p*_D3 vs. D10_ = 0.717 ***p***_**D3 vs. D90**_ **= 0.026**	44 (36–48) ***p***_**D10 vs. D90**_ **= 0.003**	48 (44–48)

*p-values in bold are statistically significant; pSSS- Scandinavian Stroke Scale prognostic score; dSSS-Scandinavian Stroke Scale long term score.*

We found a correlation between the percentage of CD4+ or CD8+ T cells and clinical status assessed with SSS. The percentage of CD4+ T cells on D1 positively correlated with clinical status assessed with SSS on D1, D3, and D10. The percentage of CD4+ T cells on D3 and D10 positively correlated with clinical status assessed with SSS on D1 and D3. At the same time points, CD8+ T cells showed an opposite correlation (Table 7). The CD4+/CD8+ ratio on D1 positively correlated with clinical status on D1, D3, and D10, while the CD4+/CD8+ ratio on D3 and D10 positively correlated with clinical status on D1 and D3. This indicates that a higher CD4+/CD8+ ratio correlated with better clinical status during the acute and subacute phases of stroke. No statistically relevant correlation between CD4−CD8− T cells and clinical status was revealed.

**Table 7:** Correlations between neurologic and functional status and CD4/CD8 populations.

	dSSS D1		dSSS D3		dSSS D10	
	rS	*p*	rS	*p*	rS	*p*
CD4 D1	**0.41**	**<0.01**	**0.37**	**<0.01**	**0.36**	**<0.05**
CD8 D1	**−0.31**	**<0.05**	**−0.27**	**<0.05**	**−0.28**	**<0.05**
CD4 D3	**0.34**	**<0.05**	**0.29**	**<0.05**	0.26	ns
CD8 D3	**−0.34**	**<0.05**	**−0.30**	**<0.05**	**−0.29**	**<0.05**
CD4 D10	**0.36**	**<0.05**	**0.32**	**<0.05**	0.20	ns
CD8 D10	**−0.37**	**<0.05**	−0.21	ns	−0.20	ns

Values in bold are statistically significant; dSSS-Scandinavian Stroke Scale long term score.

#### 3.2.2 SAI and the volume of infarct lesion

Twenty-five patients (48%) met the criteria for SAI. Most of them (56%) presented symptoms of pneumonia, 36% were diagnosed with urinary tract infections, and 8% had other infections. There were no differences between SAI+ and SAI− subjects as well as between SAI+ stroke patients and disease controls in the percentage and the absolute number of circulating CD4+ and CD8+ T cells at any time point after stroke. Nevertheless, the percentage of CD8+ T cells in the acute phase (D1) positively correlated with CRP levels during the acute and subacute phases of stroke (D1, D3, D10), as well as in the control group (Table 8). On the other hand, a negative correlation was noted between the percentage of CD4+ T cells on D1 and the serum level of CRP on D10 after stroke (Table 9). Similarly, the CD4+/CD8+ ratio on D1 negatively correlated with CRP on D1, D3, and D10. No association between the percentage of CD4−CD8− T cells and CRP in stroke patients and controls was found.

**Table 8:** CRP values in stroke patients and the disease controls.

	CRP	*p*
Stroke D1	6.2 (2.0–8.4)	***p***_**D1 vs. DC**_ **= 0.011** ***p***_**D3 vs. DC**_ **= 0.004** *p*_D10 vs. DC_ = 0.074 *p*_D90 vs. DC_ = 0.673 *p*_D1 vs. D3_ = 0.548 *p*_D1 vs. D10_ = 0.979 ***p***_**D1 vs. D90**_ **= 0.023** *p*_D3 vs. D10_ = 0.645 ***p***_**D3 vs. D90**_ **= 0.001** ***p***_**D10 vs. D90**_ **= 0.032**
Stroke D3	6.3 (2.8–16)	
Stroke D10	4.0 (1.4–15.7)	
Stroke D90	2.3 (0.9–5.2)	
Disease controls	1.9 (0.6–5.7)	

*p-values in bold are statistically significant.*

**Table 9:** Correlations between CRP level and CD4/CD8 populations.

	CRP D1		CRP D3		CRP D10		CRP DC	
	rS	*p*	rS	*p*	rS	*p*	rS	*p*
CD4 D1	0.21	ns	−0.21	ns	**−0.33**	**<0.05**	−0.21	ns
CD8 D1	**0.37**	**<0.01**	**0.36**	**<0.05**	**0.46**	**<0.01**	**0.37**	**<0.05**
CD4 D3	−0.16	ns	**−**0.07	ns	−0.20	ns	–	**–**
CD8 D3	0.19	ns	0.20	ns	0.25	ns	–	**–**
CD4 D10	0.06	ns	−0.08	ns	−0.05	ns	–	**–**
CD8 D10	0.09	ns	0.17	ns	0.09	ns	–	–
CD4 D90	0.13	ns	0.00	ns	0.09	ns	–	–
CD8 D90	−0.22	ns	0.01	ns	−0.14	ns	–	–

There were no associations between infarct volume measured at either D1 or D90, and the percentage of CD4+ T cells, CD8+ T cells, DNT, or the CD4+/CD8+ ratio at any time point after stroke.

#### 3.2.3 Vascular disease risk factors

**Figure 4:**
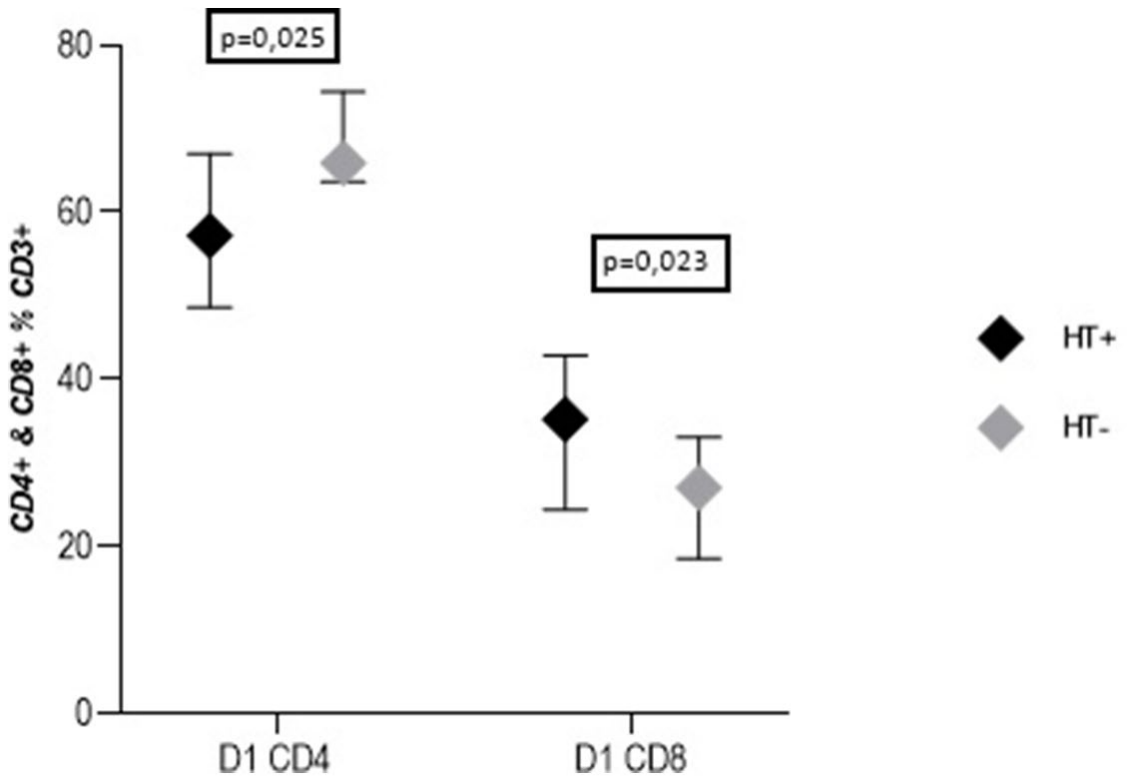
Percentage of CD4^+^ and CD8^+^ lymphocytes on day 1 of stroke in patients with hypertension in medical history (HT+) and without hypertension (HT−) (Me ± QR).

Forty-two (81%) of the patients were diagnosed with hypertension before stroke. They presented a higher percentage of CD8+ T cells and a lower percentage of CD4+ T cells in the serum than patients without a history of hypertension [CD8+ T cells: 35 (24–43) % vs. 27 (19–33) %, *p* = 0.023; CD4+ T cells: 57 (49–67) % vs. 66 (64–74) %, *p* = 0.025] (Figure 4). Such a difference was not found in the disease controls. There were no differences in the CD4+ and CD8+ T cell subpopulations regarding other vascular disease risk factors such as diabetes, hyperlipidemia, atrial fibrillation, or smoking in both studied groups.

## 4 Discussion

Although leukocytosis and lymphocytopenia are well-known phenomena in systemic reactions to brain infarction, the biological significance of T cell changes in stroke is still being explored. Moreover, the CD4+/CD8+ ratio was never used as a marker of stroke patients’ status.

In the presented study, a decreased absolute number of circulating lymphocytes and a decreased percentage of lymphocytes within WBC during acute and subacute stroke phases were revealed, in comparison to the control group. The absolute number and percentage of CD4+ and CD8+ T cells in a population of CD3+ T cells neither differ in comparison to the control group nor change with the time after stroke. Similarly, T-cell lymphocytopenia, occurring within a few hours after the stroke onset has been described by Chamorro et al. (2006), Ross et al. (2007), Haeusler et al. (2008), Klehmet et al. (2009), and Adamski et al. (2014). Haeusler et al. (2008), Klehmet et al. (2009), and Vogelgesang et al. (2019) observed a rapid decrease in CD4+ T cells, together with a lack of changes within the CD8+ T cells subgroup. Adamski et al. (2014) did not observe any changes in CD4+ and CD8+ T cells, while Jiang et al. (2017) and Liesz et al. (2009b) showed a decreased number of CD4+ and CD8+ T cells in a population of CD3+ T cells in the acute stroke phase. In Hug et al. (2011) research, no changes in CD4+ after stroke were observed.

In the present study, a relevant connection between clinical status and CD4+ or CD8+ T cell percentage was shown. We found that the higher the percentage of CD4+ T cells in the acute and subacute phases of stroke, the better the clinical status, while the higher the percentage of CD8+ T cells, the worse the clinical status. In other words, a higher CD4+/CD8+ ratio related to better clinical conditions for our patients at the beginning of the stroke. A similar correlation was revealed by Vogelgesang et al. (2008), where a higher number of T CD4+ T cells was observed in patients with better clinical status in comparison to those with worse clinical status. Nevertheless, the patients examined by Tuttolomondo et al. (2015) showed an increased number of CD4+ CD28− T cells in the blood associated with stroke severity and serum levels of proinflammatory cytokines. CD4+ CD28− T cells are an interesting subset of T cells that enhance effector functions, are associated with senescent T cells, and expand under inflammatory conditions (Dumitriu, 2015). However, considering post-stroke changes in the proportion of T cells, with a predominance of CD4+ T cells, especially the neuroprotective type of CD4+ T cells—Th2 type (Prass et al., 2003) our findings may confirm the potential benefits of this phenomenon.

Moreover, we observed a positive correlation between CRP levels on D1, D3, and D10 and the percentage of CD8+ T cells on D1. This may indicate a link between CD8+ T cells and inflammation, as well as the potential detrimental role of CD8+ T cells at the very early stage of ischemia. This finding is astonishing, as CD8+ T cells are crucial in the adaptive immune response, and their harmful effects are typically seen in later phases due to their adaptive nature (Selvaraj et al., 2021). Considering the unexpected speed and readiness of the immune system to develop an adaptive response to brain damage in very early stage of stroke, we can establish a hypothesis that immunological changes exist even before the stroke onset. A potential explanation for this situation may be a clinical condition that increase the risk of stroke, such as acute or chronic inflammatory conditions. We also observed a relevant, negative correlation between the percentage of CD4+ T cells on D1 and CRP levels on D10, suggesting that a lower CD4+/CD8+ ratio contributes to inflammation development. On the other hand, this finding may imply a direct influence of CRP on specific T cell populations in stroke. This topic has been explored for years, with researchers investigating receptors for CRP on T cells or other mediating molecules (Nimmerjahn and Ravetch, 2008; Li et al., 2012; Du Clos, 2013). As described by Yoshida et al. (2020), patients with melanoma could bind CRP to CD4+ and CD8+ T cells, inhibiting the proliferation, expansion, and antigen presentation of lymphocytes. Research by Zhang et al. (2015) showed that direct binding of CRP to native CD4+ T cells affects the Th1/Th2 ratio, leading to Th1 suppression, and consequently, regulating the inflammatory response.

Our study found an increased percentage of CD8+ T cells and a decreased percentage of CD4+ T cells during the acute phase of stroke in patients with a history of hypertension (HT), compared to those without HT. The activation of the adaptive immune system in the development of HT has already been demonstrated, with CD8+ T cells identified as responsible for the detrimental effects in HT (Drummond et al., 2019). Recently, a decreased number of CD8+ T cells, with no changes in CD4+ T cell numbers, was observed in stroke patients with a history of HT and a decreased number of CD8+ T cells in patients with HT from the control group in comparison to patients without HT diagnosis (Adamski et al., 2014). In addition to changes in numbers, increased activity of CD8+ T cells, confirmed in experimental studies, highlights their pathological influence (Trott et al., 2014). This led to the conclusion that in patients with HT, activated CD8+ T cells producing proinflammatory cytokines may lead to the promotion of inflammation during the acute phase of stroke.

In the present study, we did not find any statistically significant changes, neither in the absolute number nor percentage of DNT cells, at any time after stroke. We also did not reveal any correlations between DNTs and SAI or clinical outcomes. Our findings are in opposition to Meng et al. (2019) study, where a significantly upregulated DNT population in the peripheral blood of patients with acute stroke was found, suggesting their role in ischemic stroke. The number of DNTs also increased in the brain, and infiltrating DNTs were mainly located close to microglia in the ischemic brain lesions of both stroke patients and mice. Moreover, infiltrating DNTs enhanced immune and inflammatory responses and exacerbated ischemic brain injury (Meng et al., 2019). The reasons for the discrepancies observed are not fully understood, but in both studies, the small sample sizes may limit the statistical analysis and the ability to demonstrate significant and independent correlations.

## 5 Limitations

Our findings and interpretations should considered within the context of several limitations. These include the single-center research design and small group size, making multivariate statistical analyses unachievable. Additionally, there was a disproportion in the control group with respect to vascular risk factors, such as atrial fibrillation. Laboratory tests were limited to the quantitative assessment of peripheral blood, without functional studies or cytokine matching.

## 6 Conclusion

In the prospective assessment, the percentage of CD4+ or CD8+ T cells, the CD4+/CD8+ ratio, and DNTs did not change after stroke and showed no significant difference compared to controls. A higher CD4+/CD8+ ratio correlated with better clinical status in the acute and subacute phases of stroke, suggesting a detrimental effect of CD8+ T cells and a beneficial effect of CD4+ T cells already at the early stage of ischemia. The positive correlation between the percentage of CD8+ T cells and CRP levels, along with the predominance of CD8+ T cells in patients with HT, one of the most important risk factors of stroke, may indicate their proinflammatory potential in stroke pathogenesis.

## Data Availability

The raw data supporting the conclusions of this article will be made available by the authors without undue reservation.
