# Supplementary material for: Circulating CD4+, CD8+, and double-negative T cells in ischemic stroke and stroke-associated infection: a prospective case-control study
Source: Front Cell Neurosci. 2025 Apr 24;19:1547905. doi: 10.3389/fncel.2025.1547905 (PMC12058799; doi:10.3389/fncel.2025.1547905)
Supplement: Supplementary file 1 [file Table_1.doc]

Supplemental Material 1

Flowchart depicting patient selection

161 consecutive patients with suspected stroke

**Inclusion criteria:**

- stroke symptoms

-age >40

-symptoms duration <24h

70 patients

**Exclusion criteria:**

- haemorhagic stroke -8

-TIA -9

- neoplasm -1

**research group:**

52 patients
